# Absence of mutation of the *p73* gene localized at chromosome 1p36.3 in hepatocellular carcinoma

**DOI:** 10.1038/sj.bjc.6690027

**Published:** 1999-09-24

**Authors:** M Mihara, Y Nimura, S Ichimiya, S Sakiyama, S Kajikawa, W Adachi, J Amano, A Nakagawara

**Affiliations:** Division of Biochemistry,Chiba Cancer Center Research Institute, 666-2 Nitona, Chuoh-ku, Chiba 260-8717 Japan; Department of Surgery, Shinshu University School of Medicine, 3-1-1 Asahi, Matsumoto 390-8621, Japan

**Keywords:** *p73*, hepatocellular carcinoma, *p53*, mutation, mRNA expression, 1p36

## Abstract

Accumulating evidence has demonstrated that aberration of the *p53* tumour-suppressor gene is one of the pivotal genetic events in hepatocellular carcinogenesis. Recent reports suggest that the product of hepatitis B virus (HBV) interacts with p53 and that the hepatitis C virus (HCV) core protein reduces *p53* expression. A novel *p73* gene, which is related to *p53*, has recently been identified and mapped to chromosome 1p36.3, which is a locus of multiple tumour-suppressor genes for many cancers, including hepatocellular carcinoma (HCC) and neuroblastoma. Here, we investigated mRNA expression, allelotype and mutation of *p73* in 48 HCCs obtained from untreated patients. Reverse transcriptase polymerase chain reaction (RT-PCR) revealed that *p73* mRNA was expressed ubiquitously at low levels in all the tumour tissues, as well as in the adjacent normal liver tissues. The frequency of *p73* loss of heterozygosity was observed in 20% of HCCs, but PCR-single strand conformation polymorphism (SSCP) analysis showed no mutations in the 48 tumours except for three types of polymorphisms. These results suggest that *p73* may play a role in hepatocellular carcinogenesis in a different manner from a Knudson two-hit model. The regulatory mechanism of interaction between p73 and hepatitis viruses remains to be determined. © 1999 Cancer Research Campaign


*British Journal of Cancer* (1999) **79**(1), 164–167
© 1999 Cancer Research Campaign

### Absence of mutation of the *p73* gene localized at chromosome 1p36.3 in hepatocellular carcinoma

**M Mihara[1,2], Y Nimura[1,2], S Ichimiya[1], S Sakiyama[1], S Kajikawa[2], W Adachi[2], J Amano[2] and A Nakagawara[1]**

[1]Division of Biochemistry, Chiba Cancer Center Research Institute, 666-2 Nitona, Chuoh-ku, Chiba 260-8717, Japan; [2]Department of Surgery, Shinshu University School of Medicine, 3-1-1 Asahi, Matsumoto 390-8621, Japan

**Summary** Accumulating evidence has demonstrated that aberration of the *p53* tumour-suppressor gene is one of the pivotal genetic events in hepatocellular carcinogenesis. Recent reports suggest that the product of hepatitis B virus (HBV) interacts with p53 and that the hepatitis C virus (HCV) core protein reduces *p53* expression. A novel *p73* gene, which is related to *p53*, has recently been identified and mapped to chromosome 1p36.3, which is a locus of multiple tumour-suppressor genes for many cancers, including hepatocellular carcinoma (HCC) and neuroblastoma. Here, we investigated mRNA expression, allelotype and mutation of *p73* in 48 HCCs obtained from untreated patients. Reverse transcriptase polymerase chain reaction (RT-PCR) revealed that *p73* mRNA was expressed ubiquitously at low levels in all the tumour tissues, as well as in the adjacent normal liver tissues. The frequency of *p73* loss of heterozygosity was observed in 20% of HCCs, but PCR-single strand conformation polymorphism (SSCP) analysis showed no mutations in the 48 tumours except for three types of polymorphisms. These results suggest that *p73* may play a role in hepatocellular carcinogenesis in a different manner from a Knudson two-hit model. The regulatory mechanism of interaction between p73 and hepatitis viruses remains to be determined.

**Keywords:** *p73*; hepatocellular carcinoma; *p53*; mutation; mRNA expression; 1p36.3

Hepatocellular carcinoma (HCC) is one of the most frequent human cancers throughout the world. Epidemiological studies have indicated that infection with the hepatitis B virus (HBV) or hepatitis C virus (HCV) and ingestion of foods contaminated by aflatoxins predisposes to HCC (Bressac et al, 1991; Hsu et al, 1991). Recent advances in molecular genetics have revealed that the genesis of human cancer is attributed to multiple genetic alterations, including both proto-oncogenes and tumour-suppressor genes (Vogelstein et al, 1988). This is also the case in hepatocellular carcinogenesis as suggested in animal experiments (Woodchuck HCC), which have demonstrated overexpression of a rearranged c-*myc* proto-oncogene in HCCs (Möröy et al, 1986). However, the molecular mechanisms of hepatocarcinogenesis are still largely unclear.

The *p53* tumour-suppressor gene has been shown to be mutated in HCCs at variable frequencies (0–67%) (Buetow et al, 1989; Kress et al, 1992; Coursaget et al, 1993; Nishida et al, 1993; Teramoto et al, 1994; Vesey et al, 1994; Kazachkov et al, 1996; Honda et al, 1998). The relationship between *p53* and HBV or HCV has also been found to play a central role in the regulation of carcinogenesis and proliferation of HCC (Henkler et al, 1995; Wang et al, 1995; Lunn et al, 1997; Ray et al, 1997). In addition, many studies which demonstrate loss of heterozygosity (LOH) of HCCs have recently been reported. These include chromosomal loci 1p, 1q, 4q, 5q, 8p, 9p, 9q, 11p, 13q, 14q, 16q, 17p and 17q (Tsuda et al, 1990; Ding et al, 1991; Fujimori et al, 1991; Nagai et al, 1997; Piao et al, 1997). The 1p LOH in HCCs have been reported as 33% at *D1S243*, *D1S214* and *D1S228* (Kuroki et al, 1995), 48% at *D1S160* (Yeh et al, 1994) and 80% at *D1S47* (Simon et al, 1991). Yeh et al (1994) have shown that the genetic alteration in HCC appears to cluster at the region mapped to 1p35–36, suggesting the presence of tumour-suppressor gene(s) of HCC in this particular region.

A novel gene, *p73*, encoding a protein homologous to p53 has recently been identified (Kaghad et al, 1997). The *p73* gene has a remarkable sequence similarity to *p53* in the domains of transcriptional activation, DNA-binding and oligomerization (Kaghad et al, 1997). p73 activates the transcription of p53-responsive genes such as *p21*$^{waf1/cip1}$, inhibits cell growth and induces apoptosis (Jost et al, 1997). Kaghad et al (1997) found monoallelic expression of the *p73* gene in neuroblastoma cell lines, suggesting that the gene is imprinted. In addition, the fact that *p73* has been mapped to chromosome 1p36.33 led us to investigate genetic alterations of *p73* in primary HCCs.

### MATERIALS AND METHODS

### Specimens

Primary hepatocellular carcinomas and their corresponding non-cancerous liver tissues were obtained from 48 patients who received surgical resection at the Shinshu University Hospital, Nagano, Japan. Aetiologically, 38 patients had HCV infection, six HBV infection, two alcoholic liver cirrhosis, one cirrhosis due to Budd–Chiari syndrome, and one normal liver. The tumours were untreated except that Lipiodol Ultra-Fluide (Laboratoire Guerbet, Villepinte, France) was transarterially injected for a diagnostic purpose. The tissues were frozen immediately after surgery and stored at –80°C until used. DNA was prepared with a QIAamp Tissue Kit (Qiagen, Hilden, Germany). Total RNA was isolated from 43 out of the 48 cancerous tissues and their corresponding non-cancerous tissues by the standard protocol (Chomczynski et al, 1987).

*Received 18 February 1998*
*Revised 11 May 1998*
*Accepted 13 May 1998*

*Correspondence to:* A Nakagawara, Division of Biochemistry, Chiba Cancer Center Research Institute, 666-2 Nitona, Chuoh-ku, Chiba 260-8717, Japan

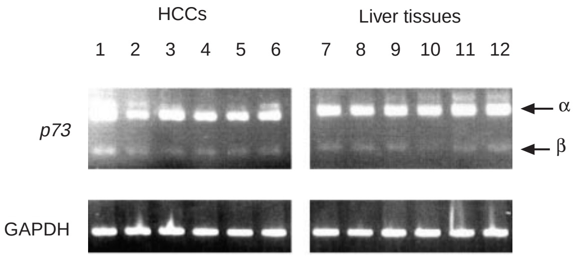

**Figure 1** Expression of *p73α* and *p73β* in hepatocellular carcinoma by RT-PCR. The PCR products, 217 bp and 113 bp, correspond in size to *p73α* and *p73β* without exon 13 respectively. Both transcripts were expressed in HCCs (lanes 1–6) and the corresponding normal liver tissues (lanes 7–12) in a similar pattern. Glyceraldehyde-3-phosphate dehydrogenase (GAPDH) was amplified as a control

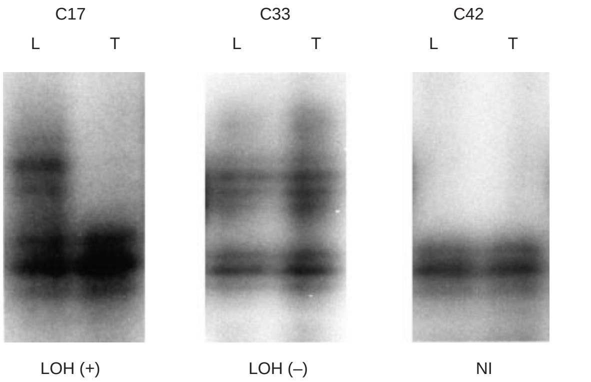

**Figure 2** *p73* loss of heterozygosity in hepatocellular carcinomas. Autoradiograghs show representatives of LOH in HCCs. DNA was isolated from the tumour (T) and the corresponding liver tissue (L). The patient's number is shown at the top of the autography. C17 had a loss of heterozygosity (LOH+), C33 showed retention of both alleles (LOH–) and the LOH of C42 was not informative (NI)

### Reverse transcriptase (RT)-PCR analysis

First-strand cDNA synthesis was performed using 2 μg of total RNA and Superscript II reverse transcriptase (Life Technologies, Rockville, MD, USA). The mRNA expression of *p73α* and *p73β* was measured by RT-PCR as described previously (Nimura et al, 1998). The primer sequences used for amplification of the polymorphic regions of the *p73* gene are shown elsewhere (Nimura et al, 1998).

### Loss of heterozygosity analysis

To study LOH of the *p73* gene, PCR reaction was performed with a primer pair flanking a CT-repeat which we found in intron 9 of the *p73* gene (Nimura et al, 1998). The primer sequences used for *p73* LOH were as follows: p73-LOH-F, 5′-CCT CTT CCT CCC CTA CCA AC-3′; and p73-LOH-R, 5′-TAG GCG ACA GAG CAA GAC G-3′. The amplification products of about 110 bp were electrophoresed on 6% denaturing polyacrylamide gels at 1500 V for 3 h.

### PCR–single-strand conformation polymorphism (SSCP) analysis

Amplification of the *p73* genomic DNA was performed using specific primer sets for each exon, which we previously designed based on the nucleotide sequence information obtained from the *p73* P1 clone (Nimura et al, 1998). To search for mutations of *p73* by PCR-SSCP analysis, exons 2–14, which cover the entire coding region of human *p73*, were amplified. Electrophoresis was carried out at room temperature at 200 V for 15 h. The gel was dried on a sheet of filter paper using a gel dryer and exposed to radiographic film at –80°C. PCR-SSCP analysis of the *p53* gene was performed according to the method described previously (Murakami et al, 1991). Three primer sets which cover the hotspot region in exons 5–9 of the *p53* gene were used.

The PCR products which showed an aberrant migration pattern on the SSCP gels were run on agarose gels and excised from the gel. The DNA fragments were purified using GenElute Agarose Spin Column (Supelco, Bellefonte, PA, USA) and subcloned into the plasmid vector (pGem-T Easy Vector, Promega, Madison, WI, USA). The individual clones were sequenced by the dideoxynucleotide termination reaction method using the ABI Prism 377 DNA sequencer (Perkin-Elmer, Foster City, CA, USA).

### RESULTS

### Expression of *p73* mRNA in HCCs

The *p73* transcripts were ubiquitously expressed in all 43 cancerous tissues and their corresponding non-cancerous liver tissues by RT-PCR as shown in Figure 1, although the transcripts were not detectable by Northern blot analysis (data not shown). In all the samples, *p73α* was expressed at higher levels than *p73β*. The semiquantitative PCR, which was done by changing the PCR cycles, showed that the levels of *p73* expression were similar in cancerous and non-cancerous tissues of the liver (data not shown). There was no significant correlation between *p73* expression and the aetiology of HCCs.

### Loss of heterozygosity of the *p73* gene in HCCs

Allelotyping of the *p73* gene was performed using DNA from the HCC tissues and the paired normal liver tissues. In 25 out of 48 (52%) HCCs, the LOH data were informative. LOH was found in five (20%) tumours (Figure 2).

### Mutation analysis of the *p73* gene in HCCs

DNA from the 48 tumours was subjected to PCR-SSCP and DNA sequencing. The DNA fragments which showed a mobility shift on the SSCP gels were sequenced, and this revealed that there were no mutations with amino acid substitution or frame shifts in the coding region of the *p73* gene. Polymorphisms at codons Ala336Ala (GCC→GCT) plus His349His (CAT→CAC), Ala 557Ala (GCG→GCA) and Ala610Ala (GCG→GCA) were found in seven (15%), four (9%) and seven (15%) respectively (Figure 3). Next, we examined the *p53* mutations using PCR-SSCP of exons 5–9, which corresponded to the DNA-binding domain. Abnormally migrating bands were observed in 4 out of the 48 case (8%) tumours.

### DISCUSSION

*p73* is a novel gene recently identified as a first family member of the *p53* tumour-suppressor gene. Like p53, a p73 protein is able to activate the transcription of p53-responsive genes, such as

© Cancer Research Campaign 1999

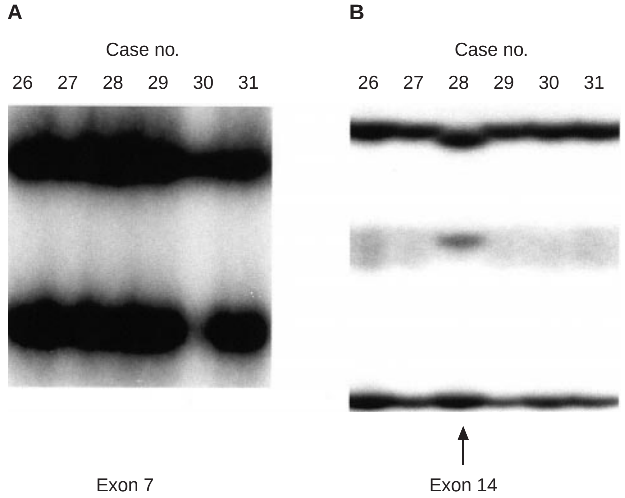

**Figure 3** PCR-SSCP analysis of *p73* in hepatocellular carcinomas. Exon 7 and exon 14 of *p73* in DNA from six hepatocellular carcinomas were PCR amplified. There were no aberrant bands in exon 7 (**A**), whereas an abnormal band shift at exon 14 was observed in C28 on the SSCP gel (**B**)

*p21*$^{waf1/cip1}$, to inhibit cell growth and to induce apoptosis (Kaghad et al, 1997; Jost et al, 1997). Because of the close similarity between p73 and p53 in both structure and function, it was suggested that *p73* might also be a tumour-suppressor gene. The hypothesis was further supported by its chromosomal localization at 1p36.3, which is a region frequently deleted in many cancers including neuroblastoma and colon cancer. In addition, Kaghad et al (1997) showed that expression of *p73* was much lower in neuroblastoma cell lines than in colorectal and breast cancer cell lines, and suggested that the *p73* gene was imprinted in neuroblastoma cell lines and normal blood leucocytes.

Our present results using RT-PCR, however, have shown that *p73* mRNA is ubiquitously expressed in HCCs. The levels of *p73* expression appear to be similar in the HCC tissues and normal liver tissues. *p73* LOH was found in 20% of HCCs. However, there were no mutations with amino acid substitution or frameshifts in the tumours. These suggest that, in HCCs, the putative *p73* tumour-suppressor gene may function in a different manner from the classical Knudson's two-hit hypothesis (Knudson, 1971). As previously suggested by Kaghad et al (1997), one of the possibilities is the imprinting of the *p73* gene. We are currently examining whether or not *p73* is imprinted in HCCs. Another possibility is the interaction of p73 with, or regulation of p73 by, the protein(s) of hepatitis viruses.

The role of p53 has been emphasized to be important in hepatocellular carcinogenesis. However, the frequency of *p53* mutations in HCC reported is variable (0–67%). Our present study showed only 8% aberrant bands on PCR-SSCP, although examination was limited to a hotspot region of exon 5–9. Wild type p53 is regulated by the proteins of hepatitis viruses which contribute to malignant transformation of hepatocytes. The HBV encodes a protein which can physically bind to p53 and apparently blocks its normal function by arresting the cell cycle (Levine et al, 1991). HBx, an oncoprotein encoded by the *X* gene on HBV DNA, can form a complex with p53 and inhibit its DNA-binding and transactivation function (Nishida et al, 1993; Henkler et al, 1995; Wang et al, 1995). A recent report has also shown that the core protein of HCV induces down-regulation of *p53* expression (Ray et al, 1997). Therefore, like p53, the p73 function could also be regulated by the protein product(s) of hepatitis viruses, although the mechanism remains to be determined.

### ACKNOWLEDGEMENTS

The authors thank Dr Shinji Nakata, Dr Yoshiro Fujimori and Dr Nobuhiko Shimozawa for collecting the HCC tissues, Dr Minoru Fujimori for his generous support, and Mrs Eunice Greenwood for proofreading the manuscript.

Support for this work was provided in part by a grant-in-aid from the Ministry of Health and Walfare for a New Comprehensive 10-Year Strategy for Cancer Control, Japan, and a grant from the Naito Foundation.

### REFERENCES

Bressac B, Kew M, Wands J and Ozturk M (1991) Selective G to T mutations of p53 gene in hepatocellular carcinoma from southern Africa. *Nature* **350**: 429–431

Buetow KH, Murray JC, Israel JL, London WT, Smith M, Kew M, Blanquet V, Brechot C, Redeker A and Govindarajah S (1989) Loss of heterozygosity suggests tumor suppressor gene responsible for primary hepatocellular carcinoma. *Proc Natl Acad Sci USA* **86**: 8852–8856

Chomczynski P and Sacchi N (1987) Single-step method of RNA isolation by acid guanidium thiocyanate–phenol–chloroform extraction. *Anal Biochem* **162**: 156–159

Coursaget P, Depril N, Chabaud M, Nandi R, Mayelo V, LeCann P and Yvonnet B (1993) High prevalence of mutations at codon 249 of the p53 gene in hepatocellular carcinomas from Senegal. *Br J Cancer* **67**: 1395–1397

Ding SF, Habib NA, Dooley J, Wood C, Bowles L and Delhanty DA (1991) Loss of constitutional heterozygosity on chromosome 5q in hepatocellular carcinoma without cirrhosis. *Br J Cancer* **64**: 1083–1087

Fujimori M, Tokino T, Hino O, Kitagawa T, Imamura T, Okamoto E, Mitsunobu M, Ishikawa T, Nakagama H, Harada H, Yagura M, Matsubara K and Nakamura Y (1991) Allelotype study of primary hepatocellular carcinoma. *Cancer Res* **51**: 89–93

Henkler F, Waseem N, Golding MHC, Alison MR and Koshy R (1995) Mutant p53 but not hepatitis B virus X protein is present in hepatitis B virus-related human hepatocellular carcinoma. *Cancer Res* **55**: 6084–6091

Honda K, Sbisa E, Tullo A, Papeo PA, Saccone C, Poole S, Pignatelli M, Mistry RR, Ding S, Isla A, Davis A and Habib NA (1998) p53 mutation is a poor prognostic indicator for survival in patients with hepatocellular carcinoma undergoing surgical tumor ablation. *Br J Cancer* **77**: 776–782

Hsu IC, Metcalf RA, Sun T, Welsh JA, Wang NJ and Harris CC (1991) Mutational hotspot in the p53 gene in human hepatocellular carcinoma. *Nature* **350**: 427–428

Jost CA, Marin MC and Kaelin Jr WG (1997) p73 is a human p53-related protein that can induce apoptosis. *Nature* **389**: 191–194

Kaghad M, Bonnet H, Yang A, Creancier L, Biscan JC, Valent A, Minty A, Chalon P, Lelias JM, Dumont X, Ferrara P, McKeon F and Caput D (1997) Monoallelically expressed gene related to p53 at 1p36, a region frequently deleted in neuroblastoma and other human cancers. *Cell* **90**: 809–819

Kazachkov Y, Khaoustov V, Yoffe B, Solomon H, Klintmalm GBG and Tabor E (1996) p53 abnormalities in hepatocellular carcinoma from United States patients: analysis of all 11 exons. *Carcinogenesis* **17**: 2207–2212

Knudson Jr AG (1971) Mutation and cancer: statistical study of retinoblastoma. *Proc Natl Acad Sci USA* **68**: 820–823

Kress S, Jahn UR, Buchmann A, Bannasch P and Schwarz M (1992) p53 mutation in human hepatocellular carcinomas from Germany. *Cancer Res* **52**: 3220–3223

Kuroki T, Fujiwara Y, Tsuchiya E, Nakamori S, Imaoka S, Knematsu T and Nakamura Y (1995) Accumulation of genetic changes during development and progression of hepatocellular carcinoma: loss of heterozygosity on chromosome arm 1p occurs at an early stage of hepatocarcinogenesis. *Genes Chrom Cancer* **13**: 163–167

Levine AJ, Momand J and Findlay CA (1991) The p53 tumor suppressor gene. *Nature* **351**: 453–456

Lunn RM, Zhang YJ, Wang LY, Chen CJ, Lee PH, Lee CS, Tsai WY and Santella RM (1997) p53 mutations, chronic hepatitis B virus infection, and aflatoxin exposure in hepatocellular carcinoma in Taiwan. *Cancer Res* **57**: 3471–3477

 *© Cancer Research Campaign 1999*

Möröy T, Marchio A, Etiemble J, Trepo C, Tiollais P and Buendia MA (1986) Rearrangement and enhanced expression of c-*myc* in hepatocellular carcinoma of hepatitis virus infected woodchucks. *Nature* **324**: 276–279
Murakami Y, Hayashi K and Sekiya T (1991) Detection of aberrations of the p53 alleles and the gene transcript in human tumor cell lines by single-strand conformation polymorphism analysis. *Cancer Res* **51**: 3356–3361
Nagai H, Pineau P, Tiollais P, Buendia MA and Dejean A (1997) Comprehensive allelotyping of human hepatocellular carcinoma. *Oncogene* **14**: 2927–2933
Nimura Y, Mihara M, Ichimiya S, Sakiyama S, Seki N, Ohira M, Nomura N, Fujimori M, Adachi W, Amano J, He M, Ping Y-M and Nakagawara A (1998) p73, a gene related to p53, is not mutated in esophageal carcinomas. *Int J Cancer* (in press)
Nishida N, Fukuda Y, Kokuryu H, Toguchida J, Yandell DW, Ikenega M, Imura H and Ishizaki K (1993) Role and mutational heterogeneity of the *p53* gene in hepatocellular carcinoma. *Cancer Res* **53**: 368–372
Piao Z, Kim H, Jeon BK, Lee WJ and Park C (1997) Relationship between loss of heterozygosity of tumor suppressor genes and histologic differentiation in hepatocellular carcinoma. *Cancer* **80**: 865–872
Ray RB, Steele R, Meyer K and Ray R (1997) Transcriptional repression of p53 promoter by hepatitis C virus core protein. *J Biol Chem* **272**: 10983–10986
Simon D, Knowles BB and Weith A (1991) Abnormalities of chromosome 1 and loss of heterozygosity on 1p in primary hepatomas. *Oncogene* **6**: 765–770
Teramoto T, Satonaka K, Kitazawa S, Fujimori T, Hayashi K and Maeda S (1994) *p53* gene abnormalities are closely related to hepatoviral infections and occur at a late stage of hepatocarcinogenesis. *Cancer Res* **54**: 231–235
Tsuda H, Zhang W, Shimosato Y, Yokota J, Terada M, Sugimura T, Miyamura T and Hirohashi S (1990) Allele loss on chromosome 16 associated with progression of human hepatocellular carcinoma. *Proc Natl Acad Sci USA* **87**: 6791–6794
Vesey DA, Hayward NK and Cooksley WGE (1994) p53 gene in hepatocellular carcinoma from Australia. *Cancer Detect Prev* **18**: 123–130
Vogelstein B, Fearon ER, Hamilton SR, Kern SE, Preisinger AC, Leppert M, Nakamura Y, White R, Smits AM and Bos JL (1988) Genetic alterations during colorectal tumor development. *N Engl J Med* **319**: 525–532
Wang XW, Gibson MK, Vermeulen W, Yeh H, Forrester K, Stürzbecher HW, Hoeijmakers JHJ and Harris CC (1995) Abrogation of p53-induced apoptosis by the hepatitis B virus X gene. *Cancer Res* **55**: 6012–6016
Yeh SH, Chen PJ, Chen HL, Lai MY, Wang CC and Chen DS (1994) Frequent genetic alterations at the distal region of chromosome 1p in human hepatocellular carcinoma. *Cancer Res* **54**: 4188–4192

*© Cancer Research Campaign 1999*
